# Dysfunction of macrophages leads to diabetic bone regeneration deficiency

**DOI:** 10.3389/fimmu.2022.990457

**Published:** 2022-10-14

**Authors:** Yufeng Shen, Yifan Zhang, Zheng Zhou, Jinyu Wang, Dong Han, Jiwei Sun, Guangjin Chen, Qingming Tang, Wei Sun, Lili Chen

**Affiliations:** ^1^ Department of Stomatology, Union Hospital, Tongji Medical College, Huazhong University of Science and Technology, Wuhan, China; ^2^ School of Stomatology, Tongji Medical College, Huazhong University of Science and Technology, Wuhan, China; ^3^ Hubei Province Key Laboratory of Oral and Maxillary Development and Regeneration, Wuhan, China; ^4^ Department of Stomatology, The First Affiliated Hospital, School of Medicine, Shihezi University, Shihezi, China

**Keywords:** macrophage, bone regeneration, diabetes mellitus, niche, dysfunction

## Abstract

Insufficient bone matrix formation caused by diabetic chronic inflammation can result in bone nonunion, which is perceived as a worldwide epidemic, with a substantial socioeconomic and public health burden. Macrophages in microenvironment orchestrate the inflammation and launch the process of bone remodeling and repair, but aberrant activation of macrophages can drive drastic inflammatory responses during diabetic bone regeneration. In diabetes mellitus, the proliferation of resident macrophages in bone microenvironment is limited, while enhanced myeloid differentiation of hematopoietic stem cells (HSCs) leads to increased and constant monocyte recruitment and thus macrophages shift toward the classic pro-inflammatory phenotype, which leads to the deficiency of bone regeneration. In this review, we systematically summarized the anomalous origin of macrophages under diabetic conditions. Moreover, we evaluated the deficit of pro-regeneration macrophages in the diabetic inflammatory microenvironment. Finally, we further discussed the latest developments on strategies based on targeting macrophages to promote diabetic bone regeneration. Briefly, this review aimed to provide a basis for modulating the biological functions of macrophages to accelerate bone regeneration and rescue diabetic fracture healing in the future.

## 1 Introduction

According to the World Health Organization (WHO), more than 422 million people are currently suffering from diabetes mellitus (DM), of whom 90% to 95% have type 2 DM (T2DM) (1–3). Among sits complications, the disruption of normal skeletal system is documented as the most common complication of T2DM (1–3). The healing times of fractures in DM patients have extended by 87% more commonly, and the risk of fracture-related complications have magnified 2.2-6.4 fold higher (3–5). Recently, bone regeneration in DM patients has attracted more attention as aging population is growing with increasing cases of metabolic diseases. The impairment of glucose and insulin metabolism results in an imbalance between osteoclastogenesis and osteogenesis fracture-related complications have magnified 2.2-6.4 fold higher (6). The accumulation of advanced glycation end products (AGEs) and the amplification cascade of reactive oxygen species (ROS) signaling promote collagen cross-linking and the generation of activated osteoclasts (7). Furthermore, the alterations caused by DM in skeletal muscle and vasculature can also affect bone regeneration. These direct or indirect effects could hinder bone metabolism and remodeling under DM conditions, leading to bone regeneration deficiency and non-healing of bone wounds (8–12). Unfortunately, the existing bone repair strategies are mostly focused on the healthy individuals, which may not be applicable to DM patients. Undoubtedly, there is an urgent need to explore novel strategies to improve bone regeneration and rescue diabetic fracture healing.

Macrophages can be found in a wide range of tissues, such as bone tissues, where they assist in maintaining homeostasis from embryonic development till adults (13, 14). Activated macrophages are generally divided into two major differentiation phenotypes, classically activated macrophages (M1-like macrophages) and alternatively activated macrophages (M2-like macrophages). M1-like macrophages play a role in pro-inflammatory response, while M2-like macrophages are mainly involved in anti-inflammatory response and tissue regeneration. Macrophages present different phenotypes and functions through their polarization in response to the changes in the microenvironment (15). Macrophages in the defect microenvironment of damage sites can precisely control immune response and osteogenesis at all stages of bone regeneration, whether the early inflammatory stage or the later repair stage (16, 17). The osteoblast function is susceptible to bone resident macrophages, as evidenced by targeted macrophage depletion which results in a reduction of osteoblastic bone formation (13, 18). The alteration of bone marrow (BM) niche in the diabetic microenvironment disrupts macrophage metabolism and functional plasticity, which prevents the macrophages switching to the pro-repair M2 like macrophages (19–22). Therefore, it is believed that strategies targeting the macrophages may help promote diabetic bone regeneration.

In this review, we elaborated the origin and function of macrophages, and the mechanism of how niche macrophages affect osteogenesis under DM conditions. Focusing on the loss of pro-regeneration of macrophages in diabetic inflammatory microenvironment, we summarized the latest developments in the strategies targeting macrophages to promote diabetic bone regeneration. This review aims to provide a basis for modulating macrophage function and behavior to improve bone regeneration and rescue diabetic fracture healing in the future.

## 2 The origin of macrophages is dependent on the environmental niches

Differences in the environmental niches within or between tissues endow macrophage subsets with the ability to coexist with unique homeostasis in different tissues (23). The heterogeneity in different tissues determines different origins of macrophages in the ecological niche.

Macrophages in brain and epidermis have the ability to renew themselves throughout life, independent of monocytes (24). Macrophages in liver, dermis, and intestine, show particular origin patterns during different developmental stages (25). For example, macrophages seeded in the intestinal mucosa from embryonic precursors show extensive proliferation *in situ* during the neonatal period, but it is almost entirely dependent on the continued replenishment of circulating monocytes in adult mice (26). In embryonic and postnatal arteries, macrophages are generated from CX3CR1^+^ precursors and BM-derived monocytes, respectively. In adulthood, the functional homeostasis of macrophages in artery is maintained by cell proliferation *via* the CX3CL1/CX3CR1 axis, rather than the recruitment of monocytes (27). Together, these findings provide a clue for the niche of macrophages in bone. The specificity of bone environmental niche and the accessibility of local niches during bone regeneration (steady-state/unsteady-state) may serve as a pivotal driver for the source of bone macrophages.

### 2.1 The origins and types of macrophages in physiological bone tissues

During the stage of embryonic hematopoiesis, HSCs move into bone to form bone tissue-resident macrophages (TRMs). TRMs in bone can proliferate modestly and the balance between osteoblast and osteoclast. The population of bone TRMs is mainly maintained by cell proliferation during adulthood, instead of recruiting monocytes (28, 29). Importantly, bone TRMs can promote angiogenesis and matrix mineralization, which is driven by bone-specific niches under the regulation of autocrine/paracrine factors, to maintain bone homeostasis (30).

Based on their own specific functions, TRMs in bone and BM can be divided into HSCs-niche macrophages, erythroblastic island macrophages, osteal macrophages (31). Especially, HSCs-niche macrophages can sense the niche signals and adjust their states accordingly, including quiescence, differentiation, self-renewal and movement (32). CXCL12 signal in the niche binds to CXCR4 on the cell membrane of HSCs-niche macrophages (33) and then maintain the quiescence of HSCs under steady state (34). Rather, granulocyte colony stimulating factor (G-CSF) signaling can promote HSCs mobilization and reduce HSCs-niche retention (35). Granulocyte-colony stimulating factor receptor (G-CSFR) bound with G-CSF in HSCs-niche macrophages and induce macrophage polarization to the M2-like subtype (36).

Osteoclasts is a classic representative of bone TRMs, which exists at all stages of bone healing (37). As a subpopulation of bone-BM tissue macrophages, embryonic erythroid progenitor (EMPs)-derived osteoclast precursors are generated separately from the HSCs lineage, and osteoclasts from EMP and HSC lineages may have the potential for cell-cell fusion (38). Osteoclasts derived from EMPs play important roles in skeletal development and tooth eruption, whereas HSCs-derived osteoclasts primarily maintain postnatal bone mass (39). Osteal macrophages derived from the hematopoietic niche, are mainly distributed in the periosteal cambium and endosteum (18). Osteal macrophages play significant roles in initiating bone healing cascades *in vivo (* 37). Osteal macrophages do not express tartrate-resistant acid phosphatase, and there is a clear difference in CD169 expression that can be used to distinguish them from osteoclasts (40). In the absence of osteal macrophages, the osteoblastic niche was disrupted and HSCs were mobilized into the blood (41).

### 2.2 The origins of macrophages in the inflammatory microenvironment

The HSCs can be activated under inflammatory conditions. Expanded HSCs have the capacity to rapidly modulate responses to inflammatory stimuli *via* the paracrine pathway (34), in which myeloid differentiation is enhanced and more monocytes are recruited (42). In the inflammatory response along with TRMs depletion, recruited monocytes can develop as macrophages and fill the empty niche left by depleted macrophages (43). Conversely, infiltrating monocytes cannot be obviously observed in inflammation without depleting TRMs, such as lipopolysaccharide (LPS)-induced peritonitis (44).

In the inflammatory microenvironment, TRMs can usually be repenished by the new macrophages differentiated from monocytes or proliferated by remaining TRMs, and then repopulate the macrophage niches (45). In traumatic inflammatory bone regeneration microenvironment, this replenishment mechanism acts as the first response of bone TRMs. After being activated, some TRMs may undergo apoptosis, leaving the empty niches available for pro-inflammatory macrophages derived from monocytes (46). Monocyte-derived macrophages secrete pro-inflammatory chemokines at the fracture sites, which contribute to microbial clearance and the amplification of local inflammatory responses, synergizing with self-proliferation of remaining TRMs to maintain bone homeostasis to promote osteogenesis and bone healing (47). In summary, the availability of niches is important for macrophages participating in bone remodeling and maintaining bone homeostasis during inflammation.

### 2.3 The origins of bone TRMs tends to be from monocytes in the diabetic microenvironment

Diabetic microenvironment (niche) is an imbalanced inflammatory microenvironment, which can interfere the physiological processes of hematopoietic stem and progenitor cells (HSPCs) and their progeny. Multiple cellular types and several inflammatory factors can show different degrees of change in diabetic BM (48).

In the diabetic niche, the number of osteoblasts is significantly reduced (49). Nestin-positive perivascular cells have also been impaired. In addition, endothelial cells (ECs) show increased oxidative stress and permeability, and reduced migratory in the diabetic niche. Together, these changes result in the deficiency of HSPCs mobilization. Beyond the changes of cellular components in the diabetic BM, intra-marrow sympathetic nerve fibers may be sparser (50). Diabetic BM displays much more pro-inflammatory cytokines, such as tumor necrosis factors-α (TNF-α), IL-1β, G-CSF and IL-3. Besides, it is reported that insulin-like growth factor 1 (IGF-1), insulin-like growth factor-binding protein 5 (IGF-5), osteoprotegerin (OPG), and vascular endothelial growth factor (VEGF), are downregulated in the diabetic BM, which impair the repopulation of HSPCs and the proliferation of ECs in BM (51). Hyperglycemia has influence on the expression of microRNAs, such as the downregulation of miR-155, regulating the homeostasis, expansion, and differentiation of stem cells (52).

Diabetes mellitus leads to the dysregulation of the entire myeloid cell lineage from progenitors to terminally differentiated cells, exhibiting the myeloid bias (53). This myeloid bias or epigenetic modification could promote more monocytes to differentiate into macrophages (54, 55). Indeed, HSPCs can also be preprogrammed to myeloid lineage by hyperglycemia *in vitro*. Myeloid progenitors from diabetic mice are more likely to differentiate into monocytes than those from the non-diabetic wild type mice (56).

**Figure 1:**
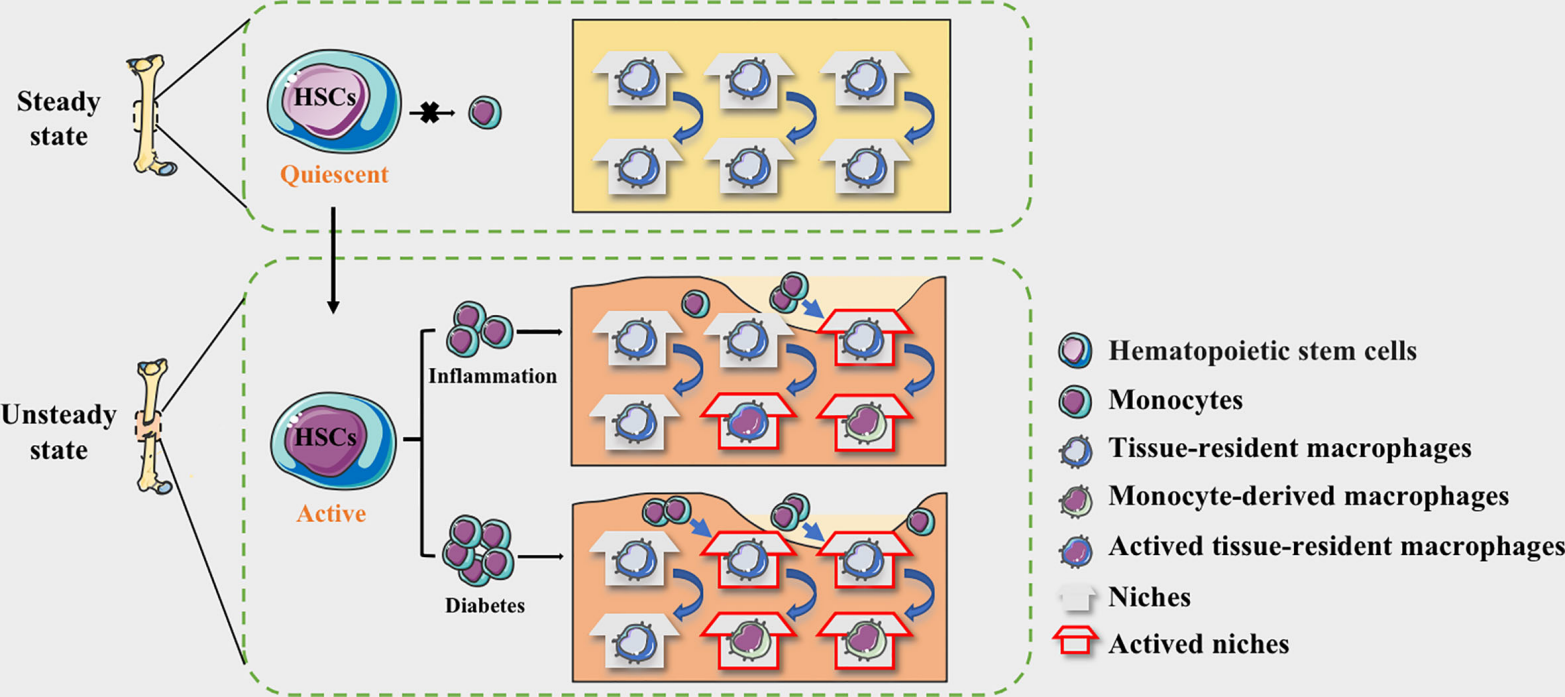
The niche of macrophages in bone tissues. HSC-derived macrophages form the tissue-resident macrophages and remain quiescent. The macrophages in the physiological bone niche are mostly inactive TRMs, maintaining homeostasis through moderate self-proliferation. HSCs could be activated by the stimulation of inflammation and the myeloid differentiation will enhance, allowing monocytes to circulate into bone tissue. The niche of resident macrophages can also be selectively activated according to the degree of inflammation, while monocyte-derived pro-inflammatory macrophages occupy available niches to maintain homeostasis. In diabetes, more available niche are occupied by pro-inflammatory macrophages, making the bone regeneration micro environment maintaining in a long-term pro-inflammatory state.

With continued exposure to the diabetic microenvironment, bone TRMs is prematurely activated (57, 58). In DM patients, dysfunctional HSPCs could induce the monocytes to mature into macrophages and acquire a prolonged pro- inflammatory phenotype, occupying the limited niche (59). The proportion of infiltrating monocyte-derived macrophages is increased, while the regenerative capacity of bone tissue resident macrophages is limited, resulting in an imbalance of niche macrophages during bone regeneration (60). Ly6C^hi^ monocytes in the blood of transplanted donors were labeled with EdU and found that blood glucose did not affect the migration of monocyte-derived macrophages from the lesions, and the reduction of EdU^+^ cells in the normal and diabetic groups was similar (51). The increased numbers of Ly6C^hi^ monocytes and circulating neutrophils are associated with common myeloid progenitors (CMPs) and granulocyte-macrophage progenitors (GMPs). Neutrophils are the predominant source of S100A8 and S100A9, and they are damage-associated molecular pattern proteins. The expression of both S100A8 and S100A9 is obviously increased in diabetic BM. Binding of S100A8/A9 to receptor for advanced glycation end products (RAGE), which is highly expressed on CMP, induces the secretion of inflammatory cytokines and the proliferation and expansion of GMP, resulting in enhanced myelopoiesis (54, 56). Together, we speculate that the source changes of macrophages in the diabetic microenvironment may be the vital cause of the persistent inflammation and damaged non-union in the process of bone regeneration (**Figure 1**).

## 3 The main roles of macrophages in bone regeneration

Affected by specific extracellular signals in the local bone regeneration microenvironment, the gene expression programs in niche macrophages are regulated by lineage-specific differentiation or specific gene expression programs, and surface marker expressions appears different, which reflect great the plasticity of macrophages (61, 62). The chromatin landscape of tissue resident macrophages in heterogeneous, leading to higher tolerance to acute inflammation, which could assist the bone homeostasis and promote the repairment (60, 63, 64). Whereas macrophages from infiltrating monocytes are more inflammatory, which mainly respond to pathological signals and participate in the innate and adaptive immune responses (65).

### 3.1 Phenotypic and functional changes of macrophages during bone healing

Macrophages work as critical cells both in physiological and pathological processes, and if they are depleted, intramembranous and endochondral osteogenesis could be significantly affected (66, 67). Macrophages with sufficient plasticity integrate multiple signals from the regenerative environment of bone tissue, and generate corresponding phenotypes and functions too adapt to niche changes (22, 68–71). In bone tissue under physiological condition, the expressions levels of CD86, CD206, macrophage colony stimulating factor (MCSF), stromal-derived factor-1(SDF-1) and CD166 in resident macrophages-osteomacs are substantially higher than those in bone marrow-derived macrophages (BMDMs). There are also significant differences in functions such as proliferation, osteoclastogenesis and phagocytosis (72). CSF-1 and IL-34 could provide essential trophic factors for survival of niche macrophages (73).

Following the repair process of bone regeneration, macrophages are polarized according to the level of extracellular factors (74). This polarization is reversible, and the change of the ecological niche makes macrophages highly dynamic, which show a diversity of responses in different stages. The accessibility and availability of niches undoubtedly determine the functional transformation of macrophages. In the early stage of bone regeneration, macrophages act as “scavengers” to remove microorganisms such as infectious substances and bacteria. At the same time, inflammatory factors such as TNF-α, IL-1β and IL-6 in the microenvironment can activate the infiltrating monocytes to differentiate into pro-inflammatory macrophages. Time and duration of inflammation significantly affect the composition of the niche and the outcome of the subsequent bone regeneration (75). In the primary callus stage, bone resident macrophages indirectly regulate the matrix microenvironment through paracrine cytokines to complete the recruitment of stem cells and their transformation to osteogenic lineage cells. The macrophages derived from monocytes gradually differentiate and mature according to the local microenvironment. Macrophages can switch between M1-like macrophages and M2-like macrophages. During bone remodeling, the secretion of pro-inflammatory factors decreases, and IL-4, IL-10, etc. can inhibit the formation of osteoclasts by inhibiting nuclear factor of activated T cells c1(NFATC1) (76, 77). The balance of the M1/M2-like macrophage niche contributes to the pro-osteogenic effect in the later stage of the repair process, which ultimately determines the quality and structure of bone.

### 3.2 Diabetes severely affects macrophage function during bone regeneration

The metabolic disturbance in diabetes coincides with changes in the number and phenotype of tissue macrophages. The metabolism of macrophages involved in bone healing is affected, both in type 1 DM (T1DM) and T2DM (78, 79). Enhanced glucose uptake and conversion to glycolysis are key features of M1-like macrophages, whereas M2-like macrophages are mainly using fatty acid oxidation and oxidative phosphorylation. Not only involved in classical inflammatory macrophage activation, glucose metabolism is also needed for alternative activation. Inhibition of glycolysis, such as 2-deoxyglucose (2-DG) can attenuate early M2 marker responses to IL-4 by decreasing oxidative phosphorylation (OxPhos) (80, 81).

#### 3.2.1 Delayed, prolonged inflammatory response

Loss of mobilization of HSPCs in diabetic BM may contribute to the insufficient and persistence inflammatory reactions (82). The deficiency of HSPCs in diabetic BM is referred to as “diabetic stem cell mobilopathy” (83). As the classical signals affecting the function and mobilization of HSPCs, excessive CXCL12 in the diabetic niche can enhance the adhesion of HSPCs to the matrix (84)and remain in their niche, unable to mobilize from the BM into peripheral blood(PB) (85). Clinical studies of BM transplantation further suggest that diabetes impairs the mobilization of HSPCs induced by G-CSF under the imbalanced state of CXCL12 in blood (84). Moreover, the altered intra-marrow sympathetic nerve fibers caused by diabetes mellitus, may reduce the flow of HSPCs and deactivate the mobilization of HSPCs induced by G-CSF (86). Compared with non-T2D patients, the level of CD34^+^ HSPCs in PB of T2DM is significantly reduced by 30-40% (87). CD45.2 BM cells from diabetic mice also exhibited a significantly lower engraftment and repopulation capacity as compared to the cells from the healthy mice (51).

Cytokine dysregulation in diabetic BM has significant effect on the induction of a protracted or delayed inflammatory response. Dysfunctional HSPCs in the diabetic BM can recruit much more monocytes and differentiate into M1-like macrophages, which release varying and numerous of pro-inflammatory factors (88). Morey, et al. reported that accumulated M1-like macrophages can express at least two-fold pro-inflammatory factors and chemokines in the *in vitro* high-glucose environment, including TNF-α, IL-1α, IL-1ß, IL-6, IL-24, colony stimulating factor 2(CSF-2), leukemia inhibitory factor (LIF), CXCL1-5, CCL4 and CCL19, etc. (89). The increased levels of Toll-like receptor 2/4 (TLR2/4) in blood from T2DM patients can also activate TLRs-MyD88-NF-κB signaling. The activation of NF-κB can upregulate the expression of various cytokines, including CCL2, CCL5, CXCL10 and TNF-α (90). However, short-term exposure to high-glucose *in vitro* has been verified to cause monocytes to secrete more IL-10, which inhibits TLRs signaling and the expression of CCL2 (91). Otherwise, *Bmi1* deficiency in the diabetic BM may lead to significant defect in the engraftment and repopulation of myeloid progenitor cells (51). Bmi1‐knockdown BMDMs can increase the expression level of IL-10, which promotes the formation of M2-like macrophages (92).

Enhanced myeloid differentiation of Ly6C^hi^ monocytes in BM (53)is a hallmark of the macrophage polarization, and the predominance of pro-inflammatory M1-like macrophages represents a major component in diabetic environments. Prolonged exposure to high glucose could lead a progressive increase in cytoplasmic glucose levels, which can cause increased mitochondrial damage and a shift in monocytes/macrophages to a pro-inflammatory macrophage (91). In the diabetic BM, there is obviously increased proportion of myeloid progenitors (MyP) and circulating inflammatory monocytes (Mo) (53), accompanying with excessive inflammatory factors secretion (89). M1-like macrophages in the diabetic BM can also raise the production of Oncostatin M (OSM), which induces the expression of CXCL12 in niche stromal cells and attenuates HSPCs mobilization, forming chronic inflammatory response (93). Hyperglycemia always change the phenotype of the autophagy-lysosomal system, causing mitochondrial ROS-induced lysosomal dysfunction, which induces more M1-like macrophage polarization in diabetes state (94). Excessive ROS acts as a signaling messenger, which links the altered metabolism and phenotypic changes with the production of proinflammatory mediators, activating some important mediators of proinflammatory signaling pathway and inducing the expression of proinflammatory genes in macrophages by inducing MAPK, STAT1, STAT6 and NF-κB signaling pathways (95). The non-enzymatic glycosylation of excessive glucose in the circulation could accumulate massive AGEs, which can activate the MAPK signaling cascade and NF-κB by binding to RAGE and further contribute to increased pro-inflammatory effects (96).

#### 3.2.2 Impaired healing

**Figure 2:**
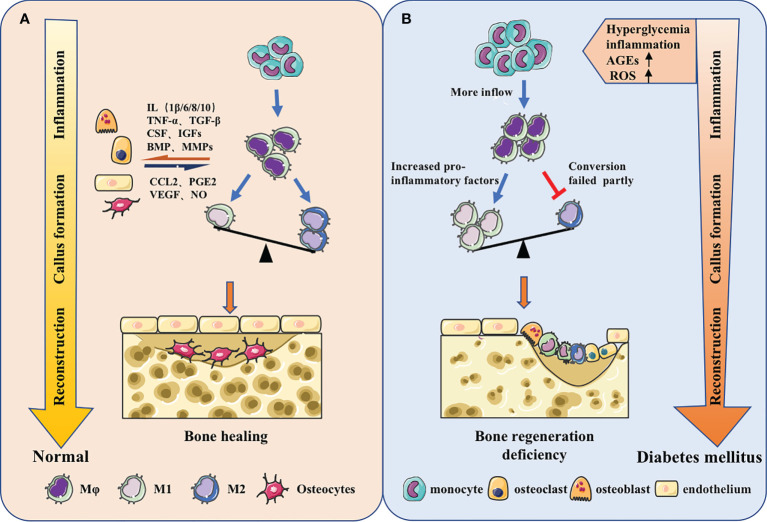
Macrophages in different bone regeneration microenvironments. **(A)** Macrophages will make adaptations in the local microenvironment. From the inflammatory stage, callus formation stage to the reconstruction stage, the predominance of pro-inflammatory M1 macrophages will gradually shift to the pro-repair M2 macrophages. Meanwhile, macrophages have coordinated cross-talk with other osteoblast-related cells, jointly regulating bone regeneration. **(B)** The origin of macrophages could change and the function could be impaired under the stimulation of high glucose, inflammation, AGEs, ROS and other metabolites in diabetes. The increase of monocyte-derived macrophages in the inflammatory phas and the failure of macrophage polarization during callus formation and bone remodeling stage could ultimately lead to insufficient osteogenesis.

Diabetes altered the macrophage plasticity during wound repair, making macrophages show hyperresponsiveness to inflammatory stimuli. The ability to switch from pro-inflammatory to pro-reparative phenotypes was impaired and the inflammatory phenotype was prolonged (97, 98). Under hyperglycemic conditions, the secondary influx of Ly6C^hi^ monocytes/macrophages delays the conversion to Ly6C^lo^ monocytes/macrophages, leading to wound healing impairment. The expression of proinflammatory and profibrotic gene is quite different between two cell types (99). At the same time, in peripheral blood, the increase of IL-1β and the drop of IFN-β in CD14^+^ monocytes could decrease JAK-STAT1 signaling, impeding macrophages’ transition to repair mode (100) (**Figure 2**).

## 4 The strategies based on macrophages for diabetic bone regeneration deficiency

Now, more and more bone regeneration schemes have turned to regulate macrophages. However, since the entire microenvironment is more complicated in diabetic patients (101), and the accessibility and availability of niches shift markedly, intervention methods used under normal physiological conditions are not yet well suited to solving the bone regeneration problems in diabetes. Therefore, proposing a more effective therapeutic strategy still faces many difficulties. Drugs, biomaterials, and a combination of both, are now frequently applied as an optimal strategy to promote bone regeneration and reconstruction by reversing the pathological state under hyperglycemia condition through modulating macrophage behavior.

### 4.1 Decrease of excessive monocyte recruitment

In diabetic conditions, the macrophage niche alters with the increase of chemokine levels. Accordingly, chemokine-chemokine receptor signaling can activate more monocytes to infiltrate to the area of bone damage. CCL2, for example, can interact with the receptor CCR2, which exists on the surface of monocyte, to promote the recruitment of monocytes (102, 103). It has been suggested that finding drugs to reduce chemokine levels and reduce excessive pro-inflammatory monocyte infiltration could therefore be one of the methods to reduce bone loss and promote bone repair. Shen et al. found that CCL2 levels were up-regulated in the periodontium of diabetic db/db mice with periodontitis, leading to increased monocyte recruitment and a positive feedback loop that enhanced and prolonged the inflammation. Oral administration of Bindarit, a CCL inhibitor that can reduce serum CCL2 levels, suppressed the excessive proinflammatory monocyte infiltration in periodontal tissue, which decreased inflammatory cytokines secretion. As a result, the alveolar bone loss was rescued and the periodontal inflammation was alleviated in Diabetes-associated periodontitis (DP) mice (104).

### 4.2 Promotion of macrophage polarization

#### 4.2.1 Activation of phenotype transition with drug treatment

Since macrophages played an important role in diabetic microenvironment, finding a drug targeting the biological behaviors and phenotypes of macrophages, could be a pretty good option. There is an imbalance between M1 and M2-like macrophages in high glucose environment. It’s reasonable to utilize the switch of macrophage to reverse the diabetes, making more M2-like macrophages occupy the limited niches and achieving reprogramming and repolarization of macrophage (105–107). Xiang et al. reported that, using Bindarit, a dipeptidyl peptidase-4 inhibitor can repolarize macrophages from M1-like macrophages to M2-like macrophages in diabetic mice, promoting angiogenesis and bone regeneration at the bone-implant interface and suggesting a potential medication for better osseointegration on the surface of titanium (Ti) implants for diabetic patients (108).

Drugs can also promote macrophage polarization by altering the metabolic microenvironment. Glucose at high concentration destroys the balance between ROS formation and antioxidant defense. The antioxidant defense system is in the inhibitory state along with the overproduction of ROS. It has been found that ROS could produce a marked effect in the induction of M1-like macrophage polarization (109), so ROS inhibitors could attempt to be used for adjusting ROS production and decrease M1-like polarization. Studies have shown that N-acetyl cysteine (NAC) could act as a ROS scavenge to reduce ROS level, partially reversing the effect of hyperglycemia on macrophage polarization and ameliorating alveolar bone loss in periodontitis in diabetic rats (110). Chen et al. indicated that the administration of PPARβ/δ agonist GW501516 can restore abnormal macrophage polarization and rectify high glucose-mediated dysfunction *via* the upregulation of Angptl4. The *in vivo* experiment indicated that PPARβ/δ agonist could decrease bone loss in diabetic mice, suggesting that PPAR agonist treatment may potentially become a novel therapeutic target for clinical therapy (111).

**Figure 3:**
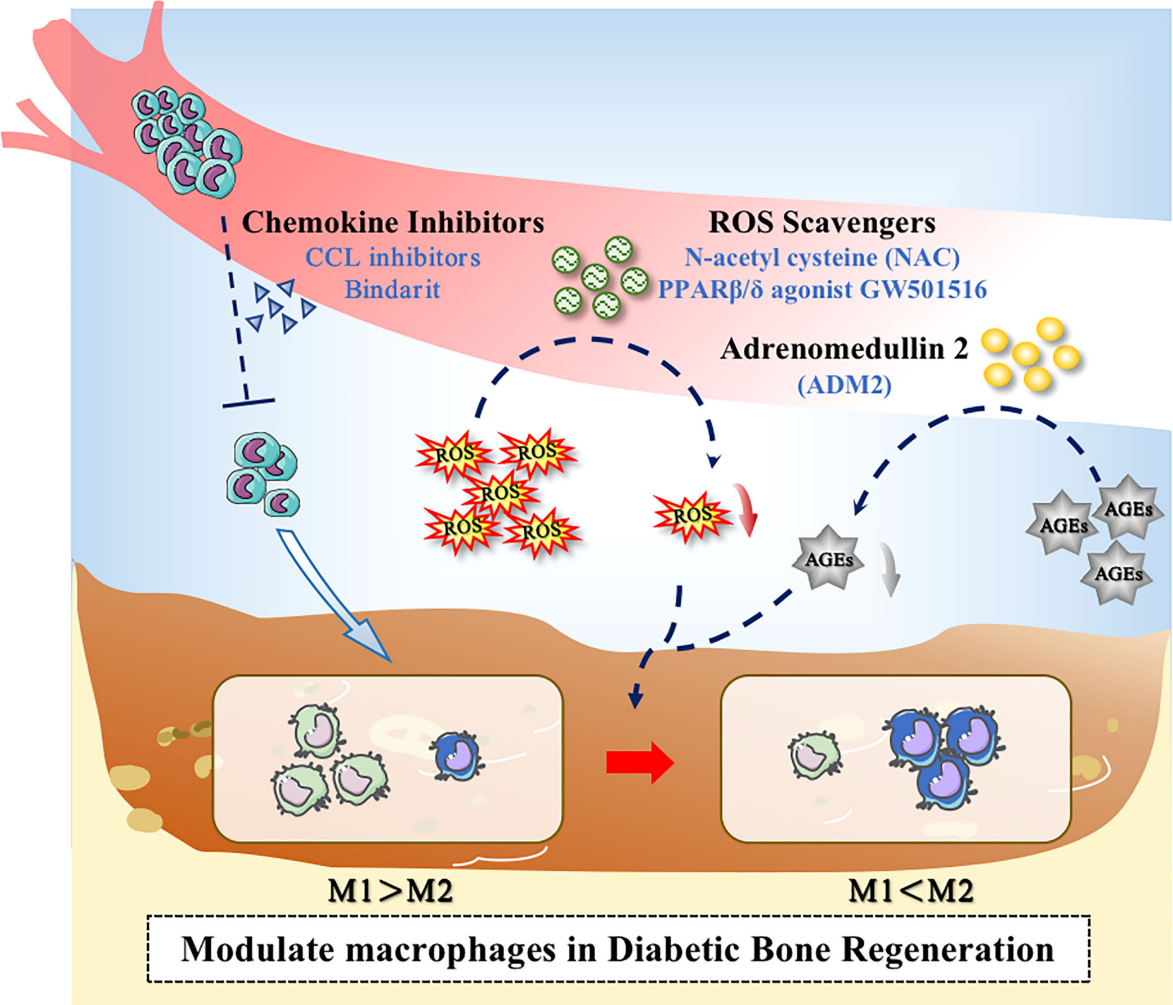
Application of drugs to modulate macrophages in the diabetic microenvironment. Drugs could promote bone regeneration in diabetes through the following ways: (1) Decreasing the excessive recruitment of monocytes; (2) Inhibiting the overproduction of ROS; (3) Inhibiting the level of AGEs.

The increased production of AGEs under hyperglycemic conditions can modulate multiple signals and affect M1-like macrophage polarization (96, 112). Studies have demonstrated that, Adrenomedullin 2(ADM2) could reverse AGE-induced macrophage inflammation and M1-like macrophage polarization *via* the PPARγ/IκBα/NF-κB signaling pathway, resulting in transformation into M2 phenotype. Applying ADM2 to a diabetic rat of Distraction osteogenesis (DO) model accelerated bone regeneration in distraction area, suggesting a new treatment for diabetic patients undergoing DO (113) (**Figure 3**).

#### 4.2.2 Stimulation of macrophages *via* drug delivery system

To develop new drug vector with good operability, adding materials and drugs together to ensure the continuous release of drug molecules, may be an efficient solution to bone regeneration. Microsphere delivery system has been investigated in a lot of studies (114, 115). Hu et al. developed an injectable microsphere, which is composed of heparin-modified gelatin nanofibers. Furthermore, interleukin 4 (IL-4) was incorporated into the microsphere (NHG-MS). As IL-4 can bind heparin, the loading efficiency of IL-4 in NHG-MS improved obviously, leading to better and more precise release of IL-4. The results suggested that the NHG-MS with encapsulation of IL-4 could restore M2/M1-like macrophages ratio to normal under DM conditions and ultimately enhanced bone regeneration (116).

Moreover, some materials can assist in achieving local release of the drug or cytokine, allowing the drug to reach an effective concentration as quickly as possible to exert more beneficial effects. Geng et al. devised an injectable silk gel scaffold loaded with sitagliptin for enhancing bone regeneration at bone-implant interface in diabetic patients. The results suggested that utilizing the scaffolds not only keep the primary action of sitagliptin in macrophage phenotypic transformation, but also enables the sustained release of selegiline *in situ* at the Ti‐bone interface, creating a local healing-promoting microenvironment and providing a new solution to implant placement failure due to diabetes (117). In another study, Chen et al. dispersed hydroxyapatite (HA) nanocrystals and magnesium oxide (MgO) nanocrystals homogeneously into the polyglutamic acid (PGA-Cys) to build a HA/MgO nanocrystal hybrid hydrogel (HA/MgO-H) scaffold. It has been reported that magnesium could decrease inflammation by switching macrophages from M1 phenotype M1-like macrophages to M2-like macrophages. The dense structure of the material maintained sufficient mechanical strength to provide mechanical support, making it hard to be washed away from the defect site, thus achieving the controlled-release of Mg^2+^ at the defect site in diabetic rats (118). Similar applications were widely utilized. For example, constructing a gelatin/β-TCP scaffold to deliver IL-4 can help promote the healing of tooth extraction socket (TES) in T2DM patients, as the IL-4 delivery system could be helpful to ameliorate the abnormal polarization (119). A recent study used gelatin, 4-arm poly (ethylene glycol) acrylate (PEG) and gelatin methacryloyl (GelMA) to prepare 3D bioprinted scaffolds, which carried RAW264.7 macrophages, bone marrow mesenchymal stem cells (BMSCs) and BMP-4-loaded mesoporous silica nanoparticles (MSNs). Owing to the better mechanical properties of the scaffolds and the application of MSNs, sustained release of BMP-4 coule be ensured. The results showed that BMP-4 promoted the polarization of M2-like macrophage. Using this new material, the repair of the calvarial critical-size defect in diabetic rat got accelerated significantly (120). Li et al. incorporated polydopamine-mediated graphene oxide (PGO) with hydroxyapatite nanoparticle (PHA) into the alginate/gelatin (AG) scaffold (121). Due to the catechol groups on the PHA and PGO, the material demonstrated advantages in antioxidation and helped prevent excessive amount of ROS. The scaffold could inhibit M1-like macrophage polarization and significantly increase M2-like macrophage polarization *via* activating the RhoA/ROCK signaling pathway, showing positive effect on periodontal bone regeneration in diabetes rat model.

#### 4.2.3 Guiding of macrophage switching by material modification

Conventional biomaterials put more emphasis on immune escape, as they were generally thought as a trigger of the immune response that has a negative impact on bone regeneration (122). Therefore, a series of inert biomaterials have been manufactured, aiming to minimize adverse immune reactions (123, 124). However, recognizing the importance of macrophage repolarization (37), studies on biomaterials have been driven to a new direction, focusing more on how to promote repolarization of macrophages. Modifying the properties of the material surface is one of the leading methods.

The properties of biomaterial surface, like morphology and wettability, can trigger different cellular response (125). It is possible to create an anti-inflammatory microenvironment through innovating material features (123, 126). Several clinical trial studies reported that implants with a hydrophilic surface showed a good survival and successful rate in patients with diabetes mellitus. Hotchkiss et al. cultured macrophages on seven different surfaces, and the results showed that micro-rough Ti lead to anti-inflammatory macrophage (M2-like) activation. Moreover, compared to hydrophobic material, the improvement of surface wettability could help produce a better microenvironment. Therefore, it can be considered as a cooperative strategy based on roughening the surface and increasing the hydrophilicity (125). The study of Lee et al. showed that the modSLA surface could promote the differentiation to M2-like macrophage while attenuating the pro-inflammatory response. It helped restore the homeostasis of the immune microenvironment, thus accelerate the osseous repair in Type 2 diabetic rats (127). Furthermore, the modSLA surface played similar role in streptozotocin-induced type I diabetic rats, which could regulate the inflammatory response in early stages and promote M2-like polarization (128).

#### 4.2.4 Modulation of macrophages through electrical microenvironment construction

**Figure 4:**
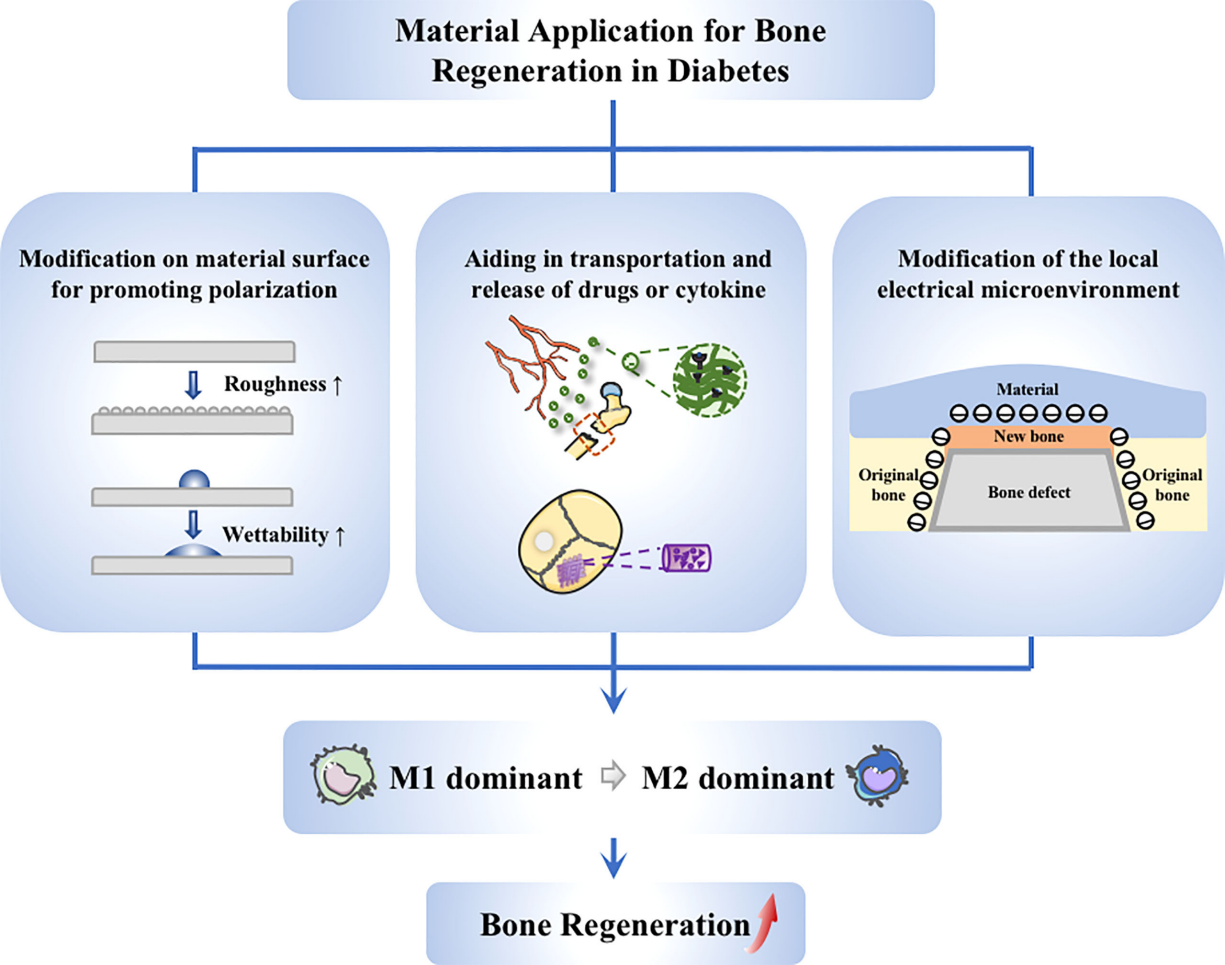
Application of materials targeting to macrophages. Strategies on material application to promote diabetic bone regeneration mainly include these aspects: (1) Guiding of macrophage switching by material modification, such as changing the surface roughness and wettability; (2) Stimulation of macrophages *via* drug delivery system, achieving better anchoring of drugs at the site of injury; (3) Modulation of macrophages through electrical microenvironment construction, creating a local pro-polarizing microenvironment to promote the transmission from M1 to M2 macrophages.

Electrical signals can regulate the functions of macrophages (129), and hence can be considered to promote macrophage phenotypic transition by using materials with good electrical properties. Dai et al. modeled electrical microenvironment inside natural bone by polarizing BaTiO3/poly (vinylidene fluoridetrifluoroethylene)(BTO/P(VDF-TrFE)) nanocomposite membrane. The polarized membrane affected the cell morphology and inflammatory cytokine secretion, which abated M1-like macrophage polarization in the hyperglycaemia states and increased the regeneration of bone in rats with type 2 diabetes mellitus. Mechanistic research indicated that the expression of AKT2 and IRF5 in the PI3K-AKT pathway was inhibited when applying the membrane, causing the hindrance of AKT2-IRF5/HIF-1α signaling (130). Considering that electrical microenvironment has a great impact on osteoimmunomodulatory and osteanagenesis, electrical characteristics become a growing interest in studies on biomaterials for guiding bone regeneration (**Figure 4**).

## 5 Summary and prospect

In recent years, there is a growing realization that alterations on the origin and phenotype of macrophages could be responsible for the persistent inflammation and impaired non-healing during diabetic bone regeneration. The niche of immune microenvironment is out of balance in the diabetic microenvironment. Indeed, some strategies targeting macrophages, applying drugs and materials, have led to some improvement in bone regeneration in diabetic conditions. However, as existing studies failed to clearly explain the molecular mechanisms in the switch of macrophages, and the interactions between macrophages of different functional phenotypes and related cells such as BMSCs and ECs were not elucidated, therapeutic options remained somewhat limited and non-specific, and the actions of relevant drugs were relatively homogeneous, making them more difficult to target macrophage during bone regeneration. It is difficult to precisely modulate bone regeneration at different stages in which macrophages are actively involved. Thus, the search for better and more effective treatment options for diabetic bone regeneration targeting macrophages is still challenging.

We believe that diabetic bone regeneration strategies targeting macrophages should gradually evolve towards multi-direction, individualization and precision. For example, new drugs should be developed so that they can not only control patients’ blood glucose levels, but also alter the abnormal microenvironment to promote bone regeneration. In addition, the physical and chemical characteristics of biomaterials need further exploration and fully utilization to establish a more precise drug release control system. The precise release of drugs for bone damage can be accomplished according to the fluctuation of specific indicators in different patients and different types of diabetes (131). Therefore, to develop an ideal treatment, there is an urgent need in basic research on the molecular mechanism of macrophage regulation and the interaction between different cells to achieve the precise regulation of macrophages in the diabetic microenvironment and reverse the imbalance of niche, which may provide more ideas and new directions for improved bone repair and regeneration in diabetic patients.

## Author contributions

YS and YZ performed the original draft preparation and revision, created the figures, and were the major contributors in writing the manuscript. ZZ, JW, and QT made suggestions to the writing of the manuscript and revisions to figures. DH, JS, and GC participated in conceptualization and methodology. QT supervised the work and critically revised the manuscript. LC and WS acquired the fundings. All authors contributed to the article and approved the submitted version.

## Funding

This work was supported by the National Natural Science Foundation of China for Key Program Projects (No. 82030070), the National Natural Science Foundation of China for Distinguished Young Scholars (No. 31725011), Hubei Provincial Natural Science Fund for Creative Research Groups (No. 2020CFA014), and Young Talents Project by the Health Commission of Hubei Province (No. WJ2021Q059).

## Conflict of interest

The authors declare that the research was conducted in the absence of any commercial or financial relationships that could be construed as a potential conflict of interest.

## Publisher’s note

All claims expressed in this article are solely those of the authors and do not necessarily represent those of their affiliated organizations, or those of the publisher, the editors and the reviewers. Any product that may be evaluated in this article, or claim that may be made by its manufacturer, is not guaranteed or endorsed by the publisher.
